# Identification of APC Mutation as a Potential Predictor for Immunotherapy in Colorectal Cancer

**DOI:** 10.1155/2022/6567998

**Published:** 2022-07-13

**Authors:** Fen Feng, Huake Sun, Zhikun Zhao, Chao Sun, Yongtian Zhao, Hanqing Lin, Jie Yang, Yajie Xiao, Wei Wang, Dongfang Wu

**Affiliations:** ^1^Cancer Center, The First People's Hospital of Foshan City, Foshan 518100, China; ^2^Department of Geriatrics, Guangzhou First People's Hospital, Guangzhou 510180, China; ^3^YuceBio Technology Co., Ltd., Shenzhen 518100, China

## Abstract

To date, anticancer immunotherapy has presented some clinical benefits to most of advanced mismatch repair deficient (dMMR)/microsatellite instability-high (MSI-H) colorectal cancer (CRC) patients. In addition to MSI status, we aimed to reveal the potential predictive value of adenomatous polyposis coli (APC) gene mutations in CRC patients. A total of 238 Chinese CRC patients was retrospectively identified and analyzed for clinical features and gene alternations in APC-mutant type (MT) and APC-wild-type (WT) groups. Clinical responses were then evaluated from the public TCGA database and MSKCC immunotherapy database. Although programmed cell death ligand 1 (PD-L1) level, MSI status, loss of heterogeneity at the human leukocyte antigen (HLA LOH), and tumor neoantigen burden (TNB) level were not statistically different between the APC-MT group and APC-WT group, tumor mutation burden (TMB) level was significantly higher in APC-MT patients (*P* < 0.05). Furthermore, comutation analysis for APC mutations revealed co-occurring genomic alterations of PCDHB7 and exclusive mutations of CTNNB1, BRAF, AFF3, and SNX25 (*P* < 0.05). Besides, overall survival from MSKCC-CRC cohort was longer in the APC-WT group than in the APC-MT group (HR 2.26 (95% CI 1.05–4.88), *P* < 0.05). Furthermore, most of patients in the APC-WT group were detected as high-grade immune subtypes (C2–C4) comparing with those in the APC-MT group. In addition, the percentages of NK T cells, Treg cells, and fibroblasts cells were higher in APC-WT patients than in APC-MT patients (*P* < 0.05). In summary, APC mutations might be associated with poor outcomes for immunotherapy in CRC patients regardless of MSI status. This study suggested APC gene mutations might be a potential predictor for immunotherapy in CRC.

## 1. Introduction

Immune checkpoint inhibitors (ICIs) targeting at the programmed cell death ligand 1 (PD-L1) or cytotoxic T lymphocyte antigen 4 (CTLA-4) signaling pathway presented impressive success in different cancer types [1–4]. Unfortunately, the overall response rate to ICI therapy remains still limited. Subsequently, some biomarkers such as PD-L1 expression, microsatellite instability (MSI), and tumor mutation burden (TMB) have been established for predicting the efficacy of immunotherapy. However, still lots of patients have low values of these recommended biomarkers. Therefore, novel diagnostic and prognostic biomarkers are expected for identifying more patients who can benefit from immunotherapy [5–7].

Colorectal cancer (CRC) ranks the third most common cancer and the second leading cause of cancer-related deaths worldwide [8]. Even though most of primary colorectal lesions are resectable, the 5-year survival rate for advanced CRC is still low. Generally, except for some patients with MSI-H/DNA mismatch repair-deficient (dMMR) tumors, CRC is supposed to be a low immune-reactive cancer with limited immune infiltrating cells or extensive infiltrating immunosuppressive T cells. In recent studies, MSI-H/dMMR CRC patients have showed lasting clinical responses and improved survival outcomes to ICI therapy [9–11]. The pity is that immunotherapy provides few clinical benefits to most of advanced non-MSI-H/dMMR CRC patients.

Adenomatous polyposis coli (APC) gene on chromosome 5q21-q22 is known as a tumor suppressor gene and is highly mutated in CRC [12, 13]. Particularly, APC mutations have demonstrated to be related with familial adenomatous polyposis (FAP) which can lead to tumor progression in CRC development [14–17]. A recent study has presented that APC is a negative regulator in the wingless signaling transduction (WNT)/beta-catenin pathway [18]. Loss of functions at APC genes can aid in proteasomal destabilization, degradation, and nuclear accumulation of beta-catenin, leading to activation of T cell factor or lymphoid enhancer factor for initiating tumorogenesis [19–22].

In addition to MSI status, we aimed to reveal the latent predictive value of APC mutations in order to provide a potential biomarker for indicating therapeutic responses in CRC patients with immunotherapy.

## 2. Methods

### 2.1. Sample Collection and Preparation

Relevant clinical and sequencing data were collected from January 2019 to June 2020, respectively. General demographic data and pathological diagnostic information were checked with corresponding medical record for each patient. A certain amount of fresh tumor tissue or formalin-fixed paraffin-embedded (FFPE) tumor tissue after a biopsy or surgery were either taken for each patient to perform PD-L1 expression analysis and genomic profiling. The study was approved by the Ethic Committee of YuceBio Technology Co., Ltd., and each patient or family member signed an informed written consent.

Genomic DNAs were isolated from each tumor tissue, and its matched peripheral blood sample was extracted using the GeneRead DNA Kit and Qiagen DNA blood mini kit (Qiagen), respectively, and extracted DNAs were then amplified, purified, and analyzed using YuceOne™ Plus NGS panel (Yucebio, China) [23, 24]. FFPE sections were stained with anti-PD-L1 22C3 mouse monoclonal primary antibody on a Dako Autostainer Link 48 system.

### 2.2. Sequencing Data Processing

Sequencing reads were filtered at the condition of >10% N rate or >10% bases with quality score <20 using SOAPnuke (Version 1.5.6). The somatic single nucleotide variants (SNVs) together with insertions and deletions (Indels) were analyzed using VarScan (Version 2.4), and furthermore, the in-house method was implemented to distinguish the possible false positive mutations. Afterwards, SnpEff (Version 4.3) was used to perform functional annotation for detected mutations in the tumor samples.

Tumor mutation burden (TMB) was measured as the total number of nonsilent somatic mutations including coding base substitution and indels per megabase, while tumor neoantigen burden (TNB) was calculated as the total number of all mutations which may generate neoantigens per megabase. HLA genotyping was assessed by OptiType [25] (Version 1.3.2), and the loss of heterogeneity (LOH) of HLA was detected as previously described [26, 27]. The levels of microsatellite instability (MSI) were calculated using the MSIsensor [28].

### 2.3. Data Acquisition and Immune Signature Analysis from Public Database

TCGA-CRC cohort data with somatic nucleotide mutations (SNVs), copy number variations (CNVs), mRNA expressions, clinical features, and survival information were downloaded from The Cancer Genome Atlas (TCGA) public database (https://portal.gdc.cancer.gov/). MSKCC pancancer or CRC immunotherapy cohort data with SNVs, clinical features, and survival information were downloaded from the cBioPortal (https://www.cbioportal.org) public database. Patients without follow-up information and survival data were excluded.

The immune subtypes were characterized into 6 immune subtypes of IFN-*γ* dominant, TGF-*β* dominant, inflammatory, lymphocyte depleted, wound healing, and immunologically quiet [29]. These subtypes were classified by macrophage or lymphocyte signature difference, Th1 cell/Th2 cell ratio, intratumoral heterogeneity level, aneuploidy, neoantigen load level, overall cell proliferation, immunomodulatory gene expression, and prognosis. Then, the proportions of infiltrating immune cells were calculated using the xCell method integrating gene set enrichment approaches with deconvolution approaches [30]. This method can provide gene signatures for 64 cell types generated from extensive expression profiles, including multiple adaptive and innate immunity cells, epithelial cells, hematopoietic progenitors, and extracellular matrix cells.

### 2.4. Statistical Analysis

Correlations between APC mutations and clinical parameters of CRC patients in this study were examined using Fisher's exact test for categorical variables. Kruskal–Wallis rank sum tests or Wilcox rank sum tests were used for comparisons of continuous variables among different groups. Survival analysis was performed from the public TCGA-CRC dataset, public MSKCC pancancer immunotherapy dataset, and MSKCC-CRC immunotherapy dataset using the Kaplan–Meier survival curve and log-rank test. *P* < 0.05 was regarded to be statistically significant. All statistical analyses in this study were performed with *R* statistical computing environment v3.6.1 software (https://www.r-project.org).

## 3. Results

### 3.1. Clinical Characteristics

As given in Table 1, a total of 238 Chinese CRC patients were identified in this study. The median age of the whole population was 59 and 58.4% (139/238) was male patients. The number of patients at grade IV was 112 (47.06%). Altogether, there were 36 patients lacking detailed information of cancer type. The number of patients with colon cancer was 113 (47.78%), while the number of patients with rectum cancer was 89 (37.39%). In general, the number of the APC-mutant type (MT) and APC-wild-type (WT) patients with CRC was 175 (73.53%) and 63 (26.37%), respectively. Besides, 93.70% (223/238) was at MSI-L status, while 6.30% (15/238) was at MSI-H status. Furthermore, there were no significant differences of gender, tumor stage, and tumor subtype between the APC-MT group and the APC-WT group. Although PD-L1, MSI, HLA LOH, and TNB were not statistically different between the APC-MT and APC-WT groups (*P* > 0.05), TMB level was significantly higher in APC-MT patients (*P* < 0.05).

### 3.2. Mutational Landscape

**Figure 1:**
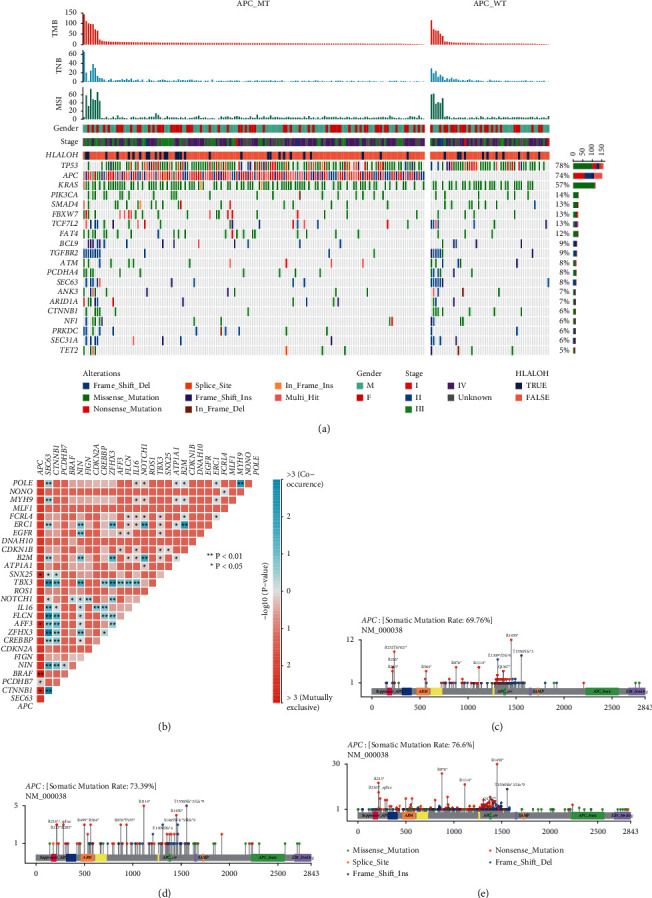
Distinct mutational patterns in Chinese CRC patients by APC gene mutation. (a) The mutational landscape of top mutated genes. (b) Co-occurring and exclusive mutations. (c) Hotspot in APC genes in NGS results. (d) Hotspot in APC genes in the public MSKCC-CRC dataset. (e) Hotspot in APC genes in the public TCGA-CRC dataset.

The 20 most frequent genomic alternations in CRC patients are shown in Figure 1(a), including TP53, APC, KRAS, PIK3CA, SMAD4, FBXW7, TCF7L2, and FAT4 with a frequency more than 10%. Furthermore, comutation analysis in Figure 1(b) revealed co-occurring genomic alterations of PCDHB7 and exclusive mutations of CTNNB1, BRAF, AFF3, and SNX25 (*P* < 0.05) with APC genes. In TCGA cohort, shown in Supplementary Figure S1, mutations of AFF3 and SNX25 were not exclusive from APC mutations (*P* < 0.1), while those alternations of CTNNB1 and BRAF were positively exclusive (*P* < 0.05). In the MSKCC database, shown in Supplementary Figure S1, there was also an indistinct mutually exclusion of CTNNB1 and BRAF from APC mutations (*P* < 0.1). Additionally, hotspot in APC genes in NGS results (Figure 1(c)) were similar with MSKCC (Figure 1(d)) and TCGA public datasets (Figure 1(e)).

### 3.3. Biomarker Analysis

**Figure 2:**
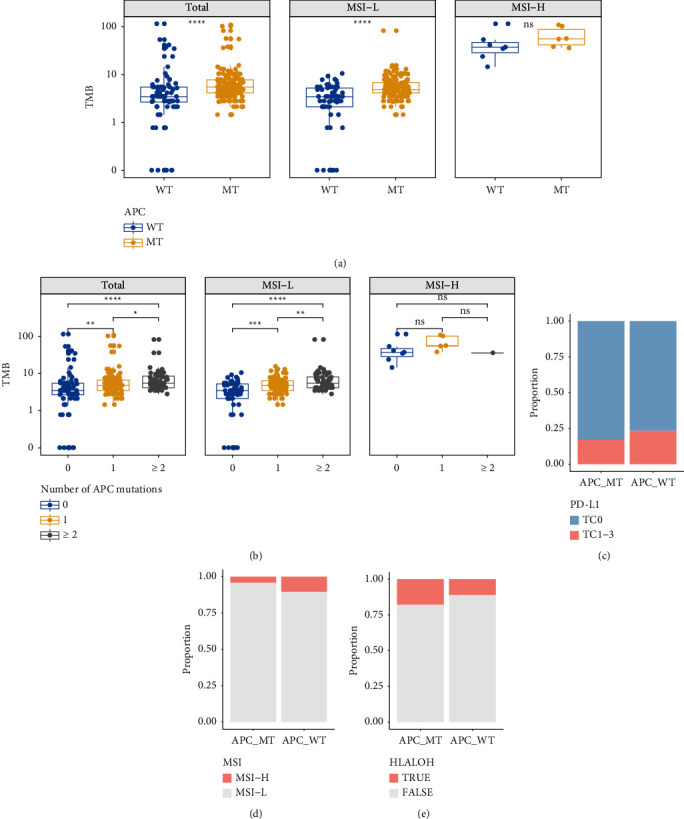
Correlation analysis in the groups with or without APC mutations. (a) TMB level with different MSI status. (b) TMB level with different MSI status and different APC mutated genes. (c) PD-L1 expression. (d) MSI status. (e) HLA LOH status. Comparisons were done by the Wilcox test or Fisher's test. N.S. was regarded to be nonstatistically significant.

Next, we also performed further analyses between other immunotherapy biomarkers and APC mutations in this study (Figures 2(a) and 2(b)). In MSI-L CRC patients, TMB level was highly correlated with APC mutations (*P* < 0.05). Unfortunately, due to small sample size, the relationship with TMB level and APC mutations was not found in MSI-H CRC patients. Besides, APC mutations were independent factors from MSI status, PD-L1 expression, and HLA LOH in this study (Figures 2(c)–2(e)).

### 3.4. Survival Analysis

**Figure 3:**
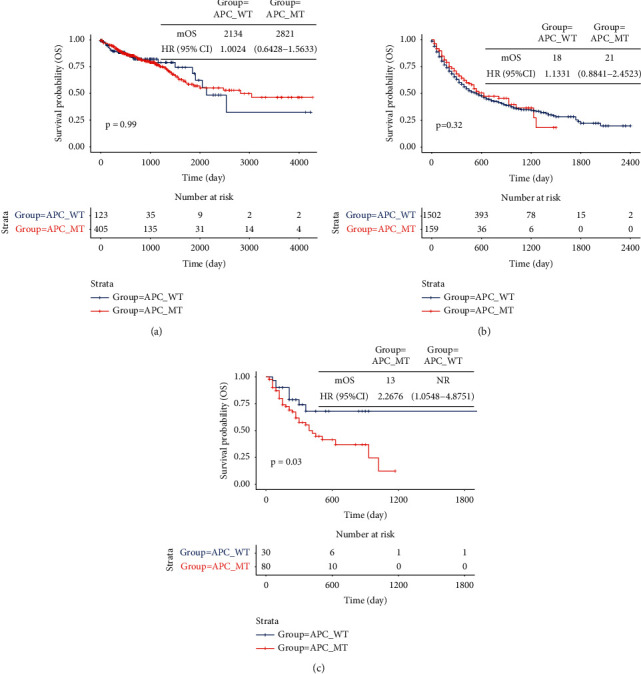
Survival analysis in the groups with or without APC mutations from public cohorts. (a) TCGA-CRC cohort. (b) MSKCC pancancer immunotherapy cohort. (c) MSKCC-CRC immunotherapy cohort.

As shown in Figures 3(a) and 3(b), patients without APC mutations did not have prolonged overall survival from TCGA CRC cohort (HR 1.00 (95% CI 0.64–1.56), *P* > 0.05) and MSKCC pancancer immunotherapy cohort (HR 1.13 (95% CI 0.88–1.45), *P* > 0.05). Interestingly, overall survival from MSKCC-CRC immunotherapy cohort was longer in the APC-WT group than in the APC-MT group (HR 2.27 (95% CI 1.05–4.88), *P* < 0.05) (Figure 3(c)).

### 3.5. Immune Signatures

**Figure 4:**
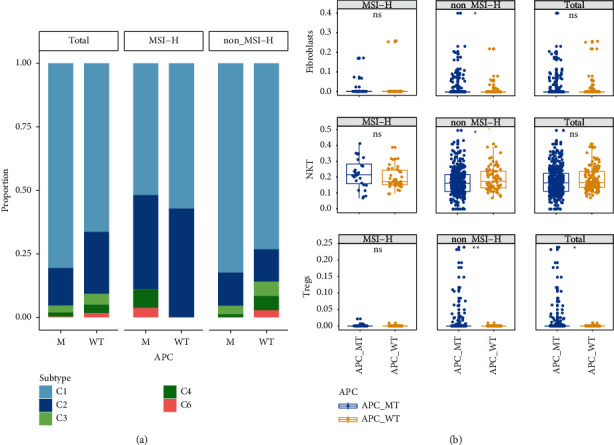
Immune signatures in the groups with or without APC mutations. (a) The percentages of immune subtypes. (b) The proportions of NK T cell, Treg cells, and fibroblasts cells. Comparisons were done by the Wilcox test or Fisher's test. N.S. was regarded to be nonstatistically significant.

Furthermore, we primarily characterized immune signatures in CRC patients in the APC-WT group and APC-MT group in Figure 4. Most of patients in the APC-WT group were detected as high-grade immune subtypes (C2–C4) compared with the APC-MT group. In addition, the percentages of NK T cell, Treg cells, and fibroblasts cells were higher in APC-WT patients with non-MSI-H status than in APC-MT patients with non-MSI-H status (*P* < 0.05). But the statistical differences were not observed in patients with MSI-H status.

## 4. Discussion

Instead of MSI status, we elucidated the predictive value of APC mutations for poor clinical responses to immunotherapy in CRC patients. Furthermore, we found that TMB was significantly higher in APC-MT patients than in APC-WT patients. In addition, we distinguished important co-occurring genomic alterations and exclusive mutations and illustrated immune signature for underlying potential mechanisms for its predictive role in CRC.

Although PD-L1 expression is the gold biomarker for immunotherapy, broad inconsistency of this biomarker can be resulted from the variability of immunohistochemical staining antibodies and heterogeneous expression [31, 32]. Recently, TMB has emerged as an important biomarker in immunotherapy, especially for prognosis prediction [10, 33]. In clinical practice, TMB level in some cancer types such as lung cancers and melanoma have been demonstrated to be substantially related with the clinical outcomes of immunotherapy. Although there were some disagreements on the cutoff values, FDA approved pembrolizumab monotherapy for adult and pediatric patients with unresectable or metastatic solid tumors of high TMB level ≥10 mut/Mb [32]. In this study, we found that TMB levels was higher in APC-MT patients than in APC-WT patients, in addition to MSI status. However, the overall TMB levels were around 5, which was much lower than those results in public CRC datasets. This inconstancy might be resulted from different sequencing methods or sequencing products. TCGA CRC cohort data were generated from whole exome sequencing, and MSKCC cohort data were generated from different targeted sequencing panels. Also, the cutoff values might be inconsistent between public datasets and our study. The definite relationship needed to be further studied.

Besides, this study also presented more alternations beyond TP53 and KRAS in APC-WT patients, which may drive tumor metastasis signaling in advanced cancers [34, 35]. As is known, APC genes can downregulate the WNT/beta-catenin pathway and consequently initiate tumorigenesis. CTNNB1, BRAF, AFF3, and SNX25 were enriched within the APC-WT CRC patients, suggesting a latent mechanism for activating WNT/beta-catenin. Prior studies indicated that CTNNB1 alternations were mutually exclusive with APC mutations, which may replace APC mutation to be the initiator genomic alteration in CRC development [36]. As an oncogenic gene for *β*-catenin mediated tumorigenesis, AFF3 can act on transcription and RNA splicing in some aggressive cancer [37]. On the other hand, mutations of PCDHB7 co-occurred in the APC-WT subgroup. Expression of the protocadherin genes such as PCDHB7 may reduce WNT signaling to *β*-catenin and protein expression of the stem cell marker [38]. These exclusive alternations to APC mutations might lead to more robust WNT activation and worse overall outcome.

To date, in-depth immunogenomic analyses with tumor-infiltrating lymphocytes in tumor microenvironments are proven to activate tumor immunogenicity. The enrichment of several adverse prognostic gene mutations in the Wnt signaling pathway is ubiquitous in tumorigenesis and cancer development [18]. In turn, recruitment of tumor-infiltrating T cells is reduced for mediating immune escape [39]. In this study, we also observed lower percentage of NK T cell, Treg cells, and fibroblasts cells and more high-grade immune subtypes (C2–C4) in the APC-WT group at non-MSI-H status compared with the APC-MT group. No significant differences were observed with patients at MSI-H status.

Our study involved several limitations. First, most of the patients in our studies were MSI-L, which might cause some statistical bias. Second, some of clinical diagnostic data such as cancer stage and tumor subtype were lacking, which cannot give a more in-depth analysis on the differences of the clinical characteristics. Third, due to lack of sufficient PFS and OS data in our real-world practice, we used public TCGA or MSKCC datasets to evaluate the predictive role of APC mutations on clinical response.

In summary, APC mutations are associated with poor outcomes of immunotherapy in CRC patients regardless of MSI status. Compared with APC-MT CRC tumors, APC-WT tumors presented more genomic alterations for activating the WNT signaling pathway. Our data suggest APC gene mutations might be a potential predictor to identify CRC patients who can benefit from immunotherapy.

## Figures and Tables

**Table 1:** Clinical characteristics and biomarkers of CRC patients.

	Total (*n* = 238)	APC-MT group (*n* = 175)	APC-WT group (*n* = 63)	*P* value
Age	59 (26–89)	61 (52–68)	59 (49–66)	0.189
Gender				
Female	99	72	27	0.882
Male	139	103	36	
Stage^#^				
I–III	64	45	19	0.614
IV	112	87	29	
Type^#^				
Colon	113	82	31	0.186
Rectum	89	72	17	
MSI				
MSI-H	15	9	6	0.223
MSI-L	223	166	57	
HLA LOH				
Yes	39	32	7	0.235
No	199	143	56	
TMB	6.69 (4.69–8.71)	6.7 (4.69–8.73)	5.36 (3.59–7.64)	0.022^*∗*^
TNB	2.62 (1.34–4.69)	2.68 (1.34–4.02)	2.55 (0.67–4.70)	0.766

TMB, tumor mutation burden; Mut/Mb, mutations per megabase; TNB, tumor neoantigen burden; Neo/Mb, neoantigens per megabase; HLA, human leukocyte antigen; LOH, loss of heterogeneity. ^*∗*^*P* values <0.05. ^#^Some data were missing.

## Data Availability

The datasets used and analyzed during the current study are available from the corresponding author upon request.

## Supplementary Materials

Supplementary Materials Supplementary Figure S1. Co-occurring and exclusive analysis with APC mutations from TCGA cohort (A) and MSKCC cohort.
