# Supplementary material for: Identification of APC Mutation as a Potential Predictor for Immunotherapy in Colorectal Cancer
Source: J Oncol. 2022 Jul 13;2022:6567998. doi: 10.1155/2022/6567998 (PMC9300385; doi:10.1155/2022/6567998)
Supplement: Supplementary Materials — Supplementary Figure S1. Co-occurring and exclusive analysis with APC mutations from TCGA cohort (A) and MSKCC cohort. [file 6567998.f1.pdf]

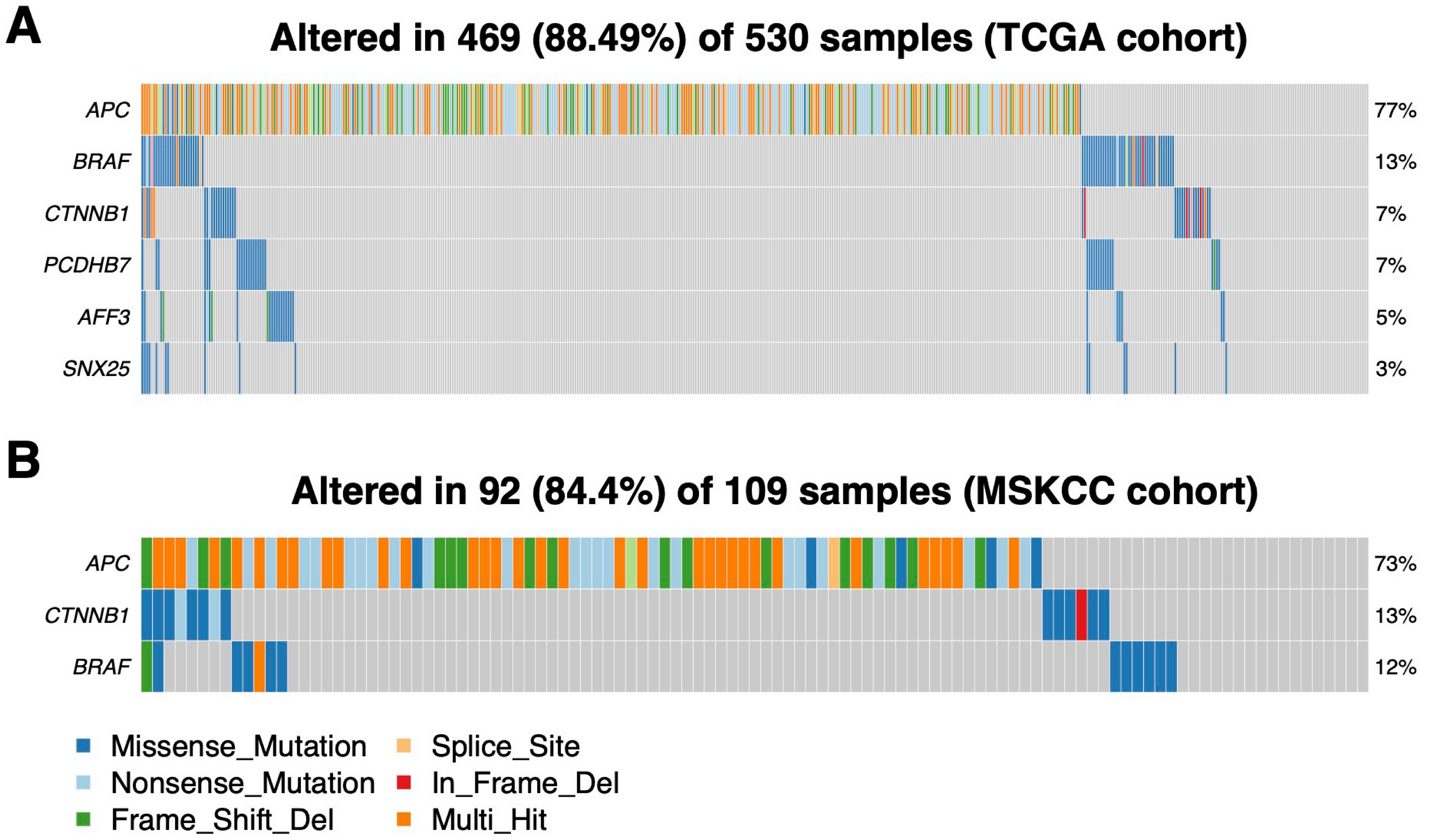
A
Altered in 469 (88.49%) of 530 samples (TCGA cohort)
APC
BRAF
CTNNB1
PCDHB7
AFF3
SNX25
77%
13%
7%
7%
5%
3%
B
Altered in 92 (84.4%) of 109 samples (MSKCC cohort)
APC
CTNNB1
BRAF
73%
13%
12%
Missense_Mutation
Nonsense_Mutation
Frame_Shift_Del
Splice_Site
In_Frame_Del
Multi_Hit
